# *Leucocytozoon cariamae* n. sp. and *Haemoproteus pulcher* coinfection in *Cariama cristata* (Aves: Cariamiformes): first mitochondrial genome analysis and morphological description of a leucocytozoid in Brazil

**DOI:** 10.1017/S0031182023000811

**Published:** 2023-12

**Authors:** Lis Marques de C. Vieira, Pedro Henrique O. Pereira, Daniel Ambrózio da Rocha Vilela, Irène Landau, M. Andreína Pacheco, Ananias A. Escalante, Francisco C. Ferreira, Érika Martins Braga

**Affiliations:** 1 Departamento de Parasitologia, Instituto de Ciências Biológicas, Universidade Federal de Minas Gerais, Belo Horizonte, MG, Brazil; 2 Centro de Triagem de Animais Silvestres, Instituto Brasileiro do Meio Ambiente e dos Recursos Naturais não Renováveis, Belo Horizonte, MG, Brazil; 3 Muséum Nation d'Histoire Naturelle, UMR7245, Molécules de Communication et Adaptation des Microorganismes, Paris, France; 4 Biology Department, Institute of Genomics and Evolutionary Medicine (iGEM), Temple University, Philadelphia, PA, USA; 5 Department of Entomology, Texas A&M University, College Station, TX, USA; 6 Schubot Center for Avian Health, Department of Veterinary Pathobiology, Texas A&M University, College Station, TX, USA

**Keywords:** avian haemosporidians, bird parasites, *cytb* gene, mtDNA genome, Neotropics

## Abstract

The distribution of avian haemosporidians of the genus *Leucocytozoon* in the Neotropics remains poorly understood. Recent studies confirmed their presence in the region using molecular techniques alone, but evidence for gametocytes and data on putative competent hosts for *Leucocytozoon* are still lacking outside highland areas. We combined morphological and molecular data to characterize a new *Leucocytozoon* species infecting a non-migratory red-legged seriema (*Cariama cristata*), the first report of a competent host for *Leucocytozoon* in Brazil. *Leucocytozoon cariamae* n. sp. is distinguished from the *Leucocytozoon fringillinarum* group by its microgametocytes that are not strongly appressed to the host cell nucleus. The bird studied was coinfected with *Haemoproteus pulcher*, and we present a Bayesian phylogenetic analysis based on nearly complete mitochondrial genomes of these 2 parasites. *Leucocytozoon cariamae* n. sp. morphology is consistent with our phylogenetic analysis indicating that it does not share a recent common ancestor with the *L*. *fringillinarum* group. *Haemoproteus pulcher* and *Haemoproteus catharti* form a monophyletic group with *Haemocystidium* parasites of Reptilia, supporting the polyphyly of the genus *Haemoproteus*. We also discussed the hypothesis that *H. pulcher* and *H. catharti* may be avian *Haemocystidium*, highlighting the need to study non-passerine parasites to untangle the systematics of Haemosporida.

## Graphical abstract

**Figure:**
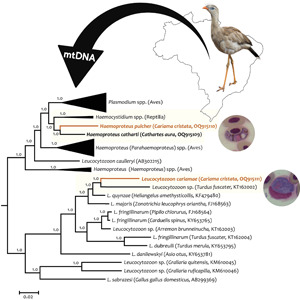

## Introduction

Vector-borne protist parasites of the order Haemosporida infect vertebrates worldwide and have clinical and ecological importance in birds (Valkiūnas, 2005; Forrester and Greiner, 2008; Pacheco and Escalante, 2023). Infections by *Haemoproteus* (*Parahaemoproteus*), family Haemoproteidae, and/or *Leucocytozoon* parasites (family Leucocytozoidae) can be lethal for captive and free-living birds (Niedringhaus *et al*., 2018; Galosi *et al*., 2019; Groff *et al*., 2019; Ortiz-Catedral *et al*., 2019; Yoshimoto, 2021). Additionally, simultaneous infections (coinfections) by these parasites may exert greater selective pressure and virulence than single infections, subsequently reducing host survival probability (Halpern and Bennett, 1983; Pigeault *et al*., 2018; Nardoni *et al*., 2020; Nourani *et al*., 2022; Pigeault *et al*., 2022).

Compared to *Haemoproteus* and *Plasmodium* (family Plasmodiidae) haemosporidians, *Leucocytozoon* parasites are less studied at global and local scales (Fecchio *et al*., 2021; Valkiūnas and Iezhova, 2023). Recent research in the Americas employing molecular techniques shows that *Leucocytozoon* infection is more frequent in high-altitude Neotropical zones (above 2200 m), such as in the Andes (Galen and Witt, 2014; Lotta *et al*., 2014, 2016, 2019; Matta *et al*., 2014).

Historically, leucocytozoid parasite transmission has been deemed negligible in the Neotropics outside these highland areas, despite the local presence of Simuliidae black flies (Figueiró *et al*., 2012; Docile *et al*., 2015; Coscarón and Coscarón-Arias, 2017; Vieira *et al*., 2017; Menzel *et al*., 2019), the Diptera insects that transmit these parasites (Valkiūnas, 2005). Early reports using microscopy alone showed low rates of *Leucocytozoon* infection in Neotropical areas (Galindo and Sousa, 1966; Forrester *et al*., 1977; White *et al*., 1978; Bennett *et al*., 1980; Bennett and Lopes, 1980; Woodworth-Lynas *et al*., 1989). However, the recent identification of diverse *Leucocytozoon* genetic lineages in blood samples from non-migratory birds across most Brazilian biomes (Fecchio *et al*., 2018, 2019; Carvalho *et al*., 2022) provides evidence of broader geographical and environmental scale transmission in the Neotropics. Such findings suggest that microscopy alone may have lower sensitivity to detect infections with low parasitaemia, called submicroscopic infections (Pacheco *et al*., 2022). *Leucocytozoon* sporozoites injected from the vector can remain viable for up to 11 days in the host (Khan *et al*., 1969) and may be amplified by polymerase chain reactions (PCRs) (Valkiūnas *et al*., 2009). Therefore, molecular-based studies cannot confirm whether the parasites detected can develop into mature gametocytes, precluding the determination of positive bird species as competent hosts (birds that are capable of transmitting the parasite to vectors) for *Leucocytozoon* in low- and mid-elevation Neotropical areas.

This study provides the first complete morphological characterization of a *Leucocytozoon* parasite infecting a non-migratory bird, the red-legged seriema (*Cariama cristata*, henceforth seriemas), in Brazil. Our thorough microscopy screening revealed a new parasite species, *Leucocytozoon cariamae* n. sp., in coinfection with *Haemoproteus pulcher*, a parasite recently described by Vanstreels *et al*. (2022). We recovered the nearly complete mitochondrial genomes (mtDNA) of the 2 parasite genera found in a single seriema using molecular cloning techniques. To improve phylogenetic inferences within *Haemoproteus* parasites, we sequenced the mtDNA genome of the MalAvi lineage CATAUR01 (Yabsley *et al*., 2018; Bensch *et al*., 2009) of *Haemoproteus catharti* from a turkey vulture (*Cathartes aura*) sampled in Pennsylvania, USA. This information was utilized to contextualize the phylogeny of these parasites within the extensive diversity of avian haemosporidians.

## Materials and methods

### Host description and sampling area

The red-legged seriema (Cariamiformes: Cariamidae) is a non-migratory sedentary species with a mean habitat range of 24 ha, which occupies open-field areas in Brazil, Bolivia, Paraguay, Argentina and Uruguay, where the humid tropical climate predominates (Souza *et al*., 2018; Winkler *et al*., 2020). The specimen from our study was rescued in the rural area of Mateus Leme (19°59′09″ S, 44°25′40″ W) municipality, Minas Gerais state, Brazil, in December of 2021 and was immediately transferred to the Wildlife Triage Center of Belo Horizonte (Centro de Triagem de Animais Silvestres – CETAS-BH). The location where the bird was rescued is a primarily urbanized mountainous territory with an average altitude of approximately 900 m dominated by sparse trees with tortuous trunks with a continuous grassy stratum, the typical vegetation of the Brazilian Cerrado *sensu stricto* (Gomes *et al*., 2016). The climate is classified as humid subtropical with dry winter and temperate summer according to Köppen's classification, with an average annual temperature of 19.3°C and precipitation of approximately 1600 mm (Alvares *et al*., 2013).

### Sampling and blood film examination

The seriema was a juvenile individual, unwilling to move, and had evidence of dog bites such as abrasions, bruises and holes in the body. Upon arrival, we physically restrained the specimen and collected 1 mL of blood through the ulnar vein. This material was used to prepare two blood smears on glass slides, which were fixed with 100% methanol for 3 min and stained with 10% Giemsa (pH = 7.2) for 70 min. The remaining blood was stored in 70% ethanol inside a refrigerator for subsequent DNA extraction and molecular analysis.

We analysed the blood smears using an Olympus CX31 microscope. Digital images were captured using an Olympus Qcolor 5 camera and processed with QCapture software. Measurements were made digitally using ImageJ (Schneider *et al*., 2012). Morphometric parameters were measured following Valkiūnas (2005), and, for the *H. pulcher* detected here, they were compared to those found in the original description (Vanstreels *et al*., 2022) using Kruskal–Wallis tests.

Intensity of infection was estimated with an initial analysis of 200 microscopic fields with monolayered blood cells at 1000× magnification. Thus, the intensity of infection was determined by counting the number of parasitized blood cells for a total of 20 000 red blood cells (Godfrey *et al*., 1987). Due to the low *Leucocytozoon* parasitaemia, we screened almost the entire extension of both blood smears to allow the morphological characterization of this parasite.

### DNA extraction, *cytb* gene amplification and sequencing

For DNA extraction, we followed the protocols described by Scopel *et al*. (2004), using a lysis buffer [50 mm NaCl, 50 mm Tris HCl (pH 7.4), 10 mm EDTA; 1% (vol/vol) Triton X-100, 200 *μ*g of proteinase K per mL] for protein lysis in a water bath for at least 18 h at 60°C, followed by the phenol–chloroform extraction method with isopropanol precipitation (Sambrook *et al*., 1989). The DNA samples were resuspended in 50 *μ*L of ultrapure water and quantified using NanoDrop 2000 (Thermo Scientific, Waltham, USA^®^) to certify the presence of DNA in adequate concentrations (40–80 ng/*μ*L) for subsequent assays.

A 4 *μ*L volume of the extraction product was used to amplify a 478 bp region of the *Leucocytozoon* spp. and *Plasmodium* spp./*Haemoproteus* spp. partial mitochondrial *cytb* gene by a nested PCR according to the protocol described by Hellgren *et al*. (2004). PCR products were visualized in a 6% polyacrylamide gel stained in silver nitrate solution.

The amplified product of the nested reaction was purified using identical volumes of polyethylene 20% glycol 6000 (Sambrook and Russell, 2001) and bi-directionally sequenced using the BigDye Terminator v3.1 Cycle Sequencing Kit (Thermo Fisher, Waltham, USA) with a volume of 10 *μ*L, including 2 *μ*L of purified product, 0.5 *μ*L of BigDye, 1.75 *μ*L of Thermo Fisher sequencing buffer, 1 *μ*L of 10 *μ*mol/ L of primer and 4.75 *μ*L of MilliQ water. We used a SimpliAmp Thermal Cycler (Applied Biosystems, Foster City, USA) for 15 s at 96°C, 15 s at 50°C and 4 min at 60°C, repeated for 30 cycles. The products were precipitated, resuspended in formamide and sequenced with dye-terminator fluorescent labelling in an ABI 3730XL sequencer (Applied Biosystems) at Institute René Rachou – Fiocruz/MG. DNA sequences were checked for the presence of mixed infections (presence of double peaks in the electropherogram), edited using ChromasPro 2.0.6 (Technelysium Pty Ltd, Helensvale, Australia) and compared with data available in the public databases GenBank (http://www4.ncbi.nlm.nih.gov) and MalAvi [Bensch *et al*. (2009), http://mbio-serv2.mbioekol.lu.se/Malavi/].

In addition to this protocol, the extracted DNA was also screened for the presence of haemosporidians using a nested-PCR protocol that targets the complete parasite *cytb* gene (1131 bp) with external (AE298 and AE299) and internal primers (AE064 and AE066) described by Pacheco *et al*. (2018*a*, 2019). Using this protocol, sequencing the PCR-amplified products from the primary and the nested PCRs confirmed the presence of *H. pulcher* (Vanstreels *et al*., 2022) in this sample but not for *Leucocytozoon* parasites.

### DNA extraction and mitochondrial genome (mtDNA) amplification

DNA was also extracted from the whole blood using the QIAamp DNA Micro Kit (Qiagen GmbH, Hilden, Germany). Nearly complete parasite mitochondrial DNA genomes (mtDNA) of *H. pulcher* and the new *Leucocytozoon* sp. found in this sample were obtained using a PCR protocol with Takara LA Taq™ polymerase (TaKaRa Takara Mirus Bio, San Jose, USA) following Pacheco *et al*. (2018*b*). In addition, to corroborate the phylogenetic relationship between *H. pulcher* and *H. catharti*, the mtDNA genome of the MalAvi lineage CATAUR01 of *H. catharti* from a turkey vulture (*C. aura*) was also amplified using the same protocol. *Haemoproteus pulcher* and *H. catharti* mtDNA genomes were amplified using the oligos forward AE170-5′ GAGGATTCTCTCCACACTTCAATTCGTACTTC 3′ and reverse AE171-5′ CAGGAAAATWATAGACCGAACCTTGGACTC 3′, and in the case of *Leucocytozoon* sp., mtDNA genome was amplified with specific oligos forward AE1130-5′ ATC AAT TGG GTT TGT GGT GGA TTT ATA ATC 3′ and AE1131-5′ AA AAC TCA TTT GAC CCC ATG GTA GG 3′. PCRs were carried out in 50 *μ*L using 4 *μ*L of the total DNA for each PCR. Negative (distilled water) and positive controls (samples from infected humans) were also included. Amplification conditions for both PCRs were a partial denaturation at 94°C for 1 min and 30 cycles with 30 s at 94°C and 7 min at 67°C, followed by a final extension of 10 min at 72°C. At least 2 independent PCR products (50 *μ*L) were excised from the gel (bands of ~6 kb), purified using the QIAquick Gel extraction kit (Qiagen, GmbH, Hilden, Germany) and cloned into the pGEM-T Easy Vector systems (Promega, Madison, USA) following the manufacturer's instructions. Both strands of 3–5 clones were sequenced at Genewiz from Azenta Life Sciences (New Jersey, USA). Inconsistencies between the clones were not found. The mtDNA genome sequences obtained in this study were identified as *Leucocytozoon* and *Haemoproteus* using BLAST (Altschul *et al*., 1990) and submitted to GenBank under accession numbers OQ915109 (*H. catharti*), OQ915110 (*H. pulcher*) and OQ915111 (*L. cariamae* n. sp.).

### Phylogenetic analysis

Phylogenetic relationships between the nearly complete mtDNA genomes of the new *Leucocytozoon* sp., *H. pulcher*, *H. catharti* and other haemosporidians were inferred on a mtDNA alignment constructed using ClustalX v2.0.12 and Muscle as implemented in SeaView v4.3.5 (Gouy *et al*., 2010) with manual editing. This alignment (5096 bp excluding gaps) included 74 partial mtDNA genome sequences belonging to 4 genera (*Leucocytozoon*, *Haemoproteus*, *Haemocystidium* and *Plasmodium*) available from GenBank (Benson *et al*., 2013) plus the 3 new sequences obtained in this study.

Then, the phylogenetic relationships were inferred on this alignment using 6 partitions (Pacheco *et al*., 2018*b*). A tree was estimated using a Bayesian method implemented in MrBayes v3.2.7 with the default priors (Ronquist and Huelsenbeck, 2003). The best model that fitted the data, as estimated by MEGA v7.0.14 (Kumar *et al*., 2016), was a general time-reversible model with gamma-distributed substitution rates and a proportion of invariant sites (GTR + Γ + I). Bayesian support was inferred for the nodes in MrBayes by sampling every 1000 generations from 2 independent chains lasting 4 × 10^6^ Markov chain Monte Carlo steps. The chains were assumed to have converged once the value of potential scale reduction factor was between 1.00 and 1.02, and the average standard deviation of the posterior probability was <0.01. Then, 25% of the samples were discarded once convergence was reached as a ‘burn-in’. GenBank accession numbers of all sequences used in this analysis are shown in the phylogenetic tree. In addition, the average evolutionary distance over all sequence pairs of *Leucocytozoon* spp., and between *H. pulcher* and *H. catharti* were estimated using the Tamura–Nei substitution model in MEGA v7.0.14 (Kumar *et al*., 2016).

## Results

### Parasite detection *via* microscopy and PCR

**Figure 1.**
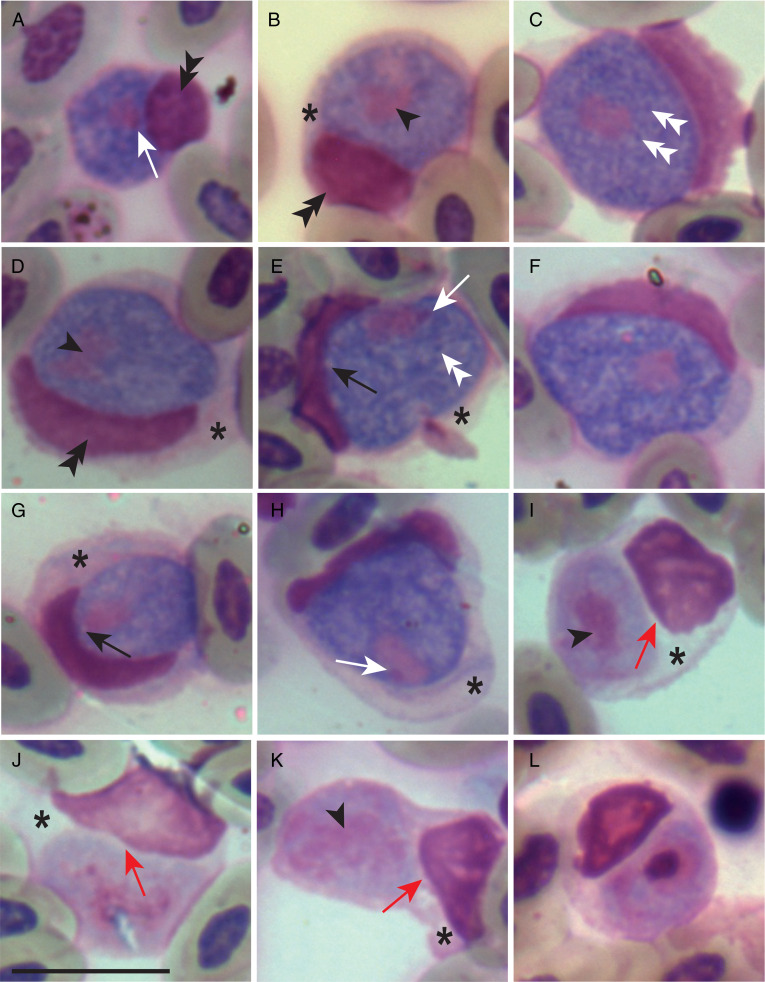
Macrogametocytes (a–h) and microgametocytes (i–l) of *Leucocytozoon cariamae* n. sp. from the blood of red-legged seriema (*C. cristata*) sampled in Brazil. Black arrowheads, parasite nucleus; double white arrowheads, volutin granules; white long arrows, parasite nucleolus; black long arrows, vacuoles; red long arrows, gap between the parasite and the host nucleus; double black arrows, host cell nucleus; asterisk, host cell cytoplasm. Giemsa-stained thin blood films. Scale bar = 10 *μ*m.

We found a single *Leucocytozoon* gametocyte in 20 000 cells analysed initially, revealing a 0.005% parasitaemia. Thus, we screened the almost entire extension of 2 blood smears and found 63 gametocytes (47 macrogametocytes and 16 microgametocytes), 18 of which were not distorted and could be included in the morphological characterization (Fig. 1; Table 1). Results from the nested PCR (Hellgren *et al*., 2004) revealed the presence of a single *Leucocytozoon* parasite previously reported in 3 seriemas captured in central Brazil (Carvalho *et al*., 2022).

**Table 1.** Morphometric parameters of mature gametocytes of *Leucocytozoon cariamae* n. sp. and its host cells from the peripheral blood of the red-legged seriema (*Cariama cristata*).

Feature	Macrogametocytes (*n* = 11)	Microgametocytes (*n* = 7)
Gametocyte		
Length	9.2–16.2 (12.6 ± 2.1)	8.5–12.9 (9.5 ± 1.5)
Width	7.7–11.0 (9.6 ± 1.3)	6.2–8.9 (7.5 ± 1.1)
Area	58.9–116.6 (96.4 ± 19.0)	40.2–74.9 (58.1 ± 12.8)
Perimeter	28.8–42.3 (37.9 ± 4.4)	24.0–33.8 (29.6 ± 3.7)
Gametocyte nucleus		
Length	2.5–4.8 (3.9 ± 0.7)	4.9–8.6 (6.1 ± 1.2)
Width	1.8–3.6 (2.6 ± 0.5)	3.0–5.0 (3.9 ± 0.7)
Area	6.1–11.3 (7.9 ± 1.8)	11.8–35.9 (21.4 ± 8.4)
Perimeter	9.1–17.4 (12.1 ± 2.4)	15.8–29.1 (22.0 ± 5.4)
Host cell–parasite complex		
Length	4.6–19.8 (14.8 ± 3.8)	8.5–15.8 (13 ± 2.4)
Width	11.5–16.8 (13.6 ± 1.7)	9.6–13.0 (10.9 ± 1.2)
Area	135.5–198.5 (168.7 ± 21.2)	101.9–155.9 (132.0 ± 21.6)
Perimeter	43.6–60.0 (50.6 ± 4.5)	38.9–56.6 (48.1 ± 5.9)
Host cell nucleus		
Circumference of parasite covered	4.7–15.3 (11.4 ± 3.2)	7.3–13.8 (10.7 ± 2.5)
Length	6.5–16.5 (12.8 ± 3.3)	8.8–15.1 (11.6 ± 2.6)
Width	1.9–4.9 (3.0 ± 1.0)	2.2–6.0 (4.4 ± 1.3)
Area	19.5–46.1 (30.4 ± 7.8)	31.9–52.3 (42.7 ± 7.6)
Perimeter	16.4–36.9 (30.2 ± 6.7)	24.1–45.4 (32.4 ± 7.6)

Minimum and maximum values are provided, followed in parentheses by the arithmetic mean and standard deviation.

**Figure 2.**
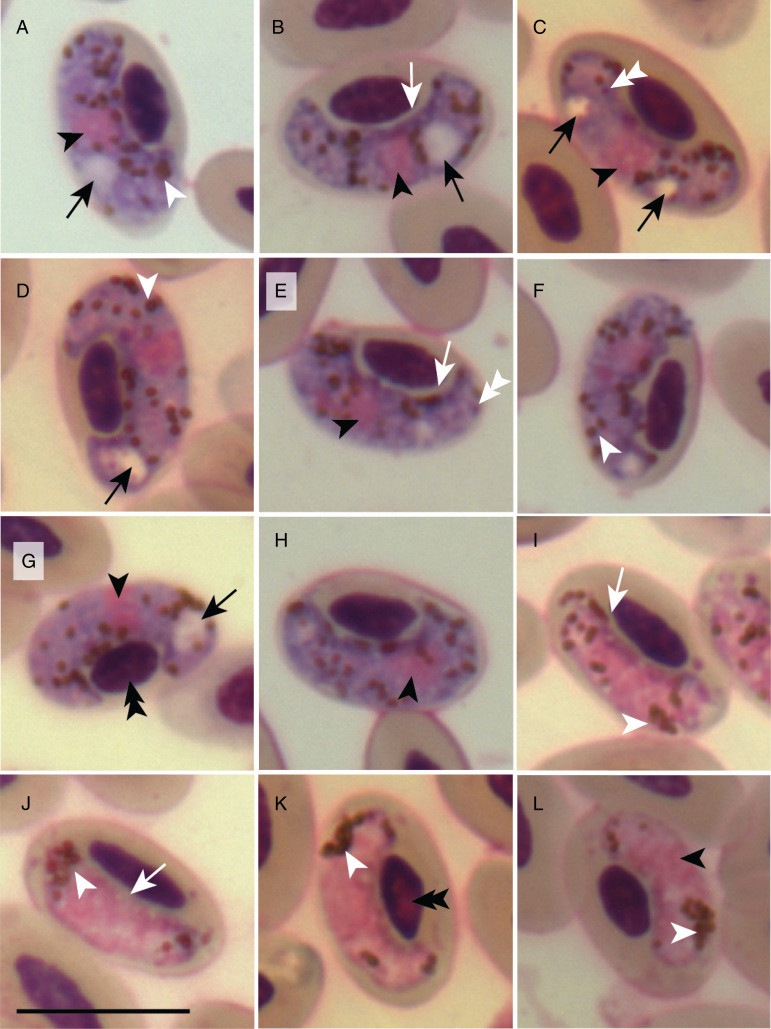
Macrogametocytes (a–h) and microgametocytes (i–l) of *Haemoproteus pulcher* from the blood of red-legged seriema (*Cariama cristata*) sampled in Brazil. Black arrowheads, parasite nucleus; white arrowheads, pigment granules; double white arrowheads, volutin granules; black long arrows, vacuoles; double black arrowheads, host cell nucleus. Giemsa-stained thin blood films. Scale bar = 10 *μ*m.

Additionally, 1.3% of the analysed erythrocytes were infected by *Haemoproteus* parasite gametocytes. This parasite displayed general characteristics similar to *H. pulcher* (Vanstreels *et al*., 2022), except for the greater number of pigment granules in microgametocytes (*P* < 0.05), the presence of volutin granules concentrated in the periphery of the parasite (Fig. 2c and e), the common presence of 1 or 2 large cytoplasmic vacuoles in macrogametocytes (Fig. 2a–d and g), the absence of gametocytes that almost encircle the erythrocyte nucleus and the absence of rounded erythrocyte nucleus in infected cells (Fig. 2; Supplementary Table S1). Additional 12 measurements showed statistically significant differences between the *H. pulcher* reported here and the original description (Supplementary Table S1), with all but 2 measurements (macrogametocyte and microgametocyte nuclei) showing greater values in our description. Sequencing the positive band obtained from the second nested-PCR protocol used in this study (Pacheco *et al*., 2018*a*) confirmed the presence of *H. pulcher*, with 100% identity in relation to the original parasite description (Vanstreels *et al*., 2022) at the *cytb* level after trimming the primer regions.

### Description of *Leucocytozoon (Leucocytozoon) cariamae* n. sp.

#### Macrogametocytes (Fig. 1a–h)

Develop in roundish host cells. Gametocytes are roundish; host cells do not produce fusiform processes. Parasite nucleus varies from rounded (Fig. 1a, b and f) to oval (Fig. 1c, e, g and h), occupying median (Fig. 1a–d) or peripheral (Fig. 1e, g and h) region. Nucleolus is visible in only 28% of the gametocytes (Fig. 1a, e, f and h). Volutin granules are abundant throughout the cytoplasm (Fig. 1c, e and f). Cytoplasmic vacuoles, when present, are small and can be mistaken for zones lacking volutin (Fig. 1e and g). The host cell nucleus is markedly displaced to the periphery and deformed into a band-like shape (Fig. 1c–h) that always extends less than half of the gametocyte's circumference (Table 1). Host cell nucleus can also be roundish (Fig. 1a and b). Variable-shaped host cell cytoplasm remnants commonly surround the gametocytes (Fig. 1b and d–g).

#### Microgametocytes (Fig. 1i–l)

Microgametocytes display a unique character of the species: the parasite does not touch or is not strongly appressed to the host cell's nucleus, always leaving a slight gap between them (Fig. 1i–l). Other than that, microgametocytes have the same general characteristics as macrogametocytes, except for common dimorphic ones, such as paler staining of the cytoplasm, a more dispersed nucleus (Fig. 1k) and smaller general size parameters (Table 1). Microgametocytes seem to have fewer vacuoles and volutin granules than macrogametocytes, although these parameters were not quantified.

#### Remarks

*Leucocytozoon cariamae* n. sp. exclusively develops in round host cells and can be easily distinguished from other leucocytozoids due to a feature present in microgametocytes, which do not or only slightly touch the host cell's nucleus. The absence of numerous vacuoles and of a prominent nucleolus in *L. cariamae* n. sp. helps to distinguish it from *Leucocytozoon dubreuili*, from the *fringillinarum* group, which typically display rounded gametocytes (Valkiūnas, 2005). As there is no convincing experimental evidence that the same *Leucocytozoon* species infects and fully develop to produce gametocytes in birds belonging to different orders (Valkiūnas and Iezhova, 2023), this first description in the order Cariamiformes establishes *L. cariamae* n. sp. as a new parasite species.

#### Taxonomic summary

***Type host:*** The red-legged seriema, *Cariama cristata* (Cariamiformes, Cariamidae).

***Additional hosts:*** Unknown.

***Type locality:*** Rural area of Mateus Leme (19°59′09″ S, 44°25′40″ W), Minas Gerais, Brazil.

***Type specimen:*** Hapantotype was deposited in the biological collection of the Muséum Nation d'Histoire Naturelle, Paris, France; the intensity of parasitaemia is 0.005%, collected by Lis Marques in the CETAS-BH, Minas Gerais, 13 December 2021.

***DNA sequences:*** The partial mitochondrial cytochrome *b* fragment (478 bp) obtained corresponded in 100% identity to the lineage CARCRI01 (GenBank acc. no. OK086053 from 3 specimens of red-legged seriemas, *C. cristata*). The nearly complete mtDNA genome (acc. no. OQ915111) corresponded to the same genetic lineage that is already deposited in GenBank (acc. no. KX832566).

***Site of infection:*** Blood cells, for which origin could not be identified due to the marked deformation caused by *L. cariamae* n. sp. gametocytes.

***Additional hosts and localities:*** Only 1 bird was evaluated in this study. However, Carvalho *et al*. (2022) found that 3 out of 3 red-legged seriemas captured in central Brazil were positive by PCR for this parasite.

***Etymology:*** The species name refers to the genus ‘*Cariama*’, the single genus of the order Cariamiformes.

### Molecular and phylogenetic analyses

#### *Cytb* gene BLAST analysis

The result of BLASTn using the partial *cytb* sequence of *L. cariamae* n. sp. as query returned 100% identity and 100% coverage in relation to the CARCRI01 lineage (OK086053) also found in seriemas sampled in the Brazilian Cerrado (Carvalho *et al*., 2022). The most closely related *Leucocytozoon* lineage reported (GenBank acc. no. KX832566) has 0.035 genetic distance in relation to *L. cariamae* n. sp. and was found in common wood pigeons (*Columba palumbus*) from the UK (see Fig. 1 in Carvalho *et al*., 2022). *Leucocytozoon cariamae* n. sp. has 0.042 genetic distance in relation to *Leucocytozoon podargii* described infecting tawny frogmouths (*Podargus strigoides*) in Australia (Jiang *et al*., 2019), and to *Leucocytozoon californicus* infecting American kestrel (*Falco sparverius*) in California, USA (Walther *et al*., 2016).

#### mtDNA phylogenetic analysis

**Figure 3.**
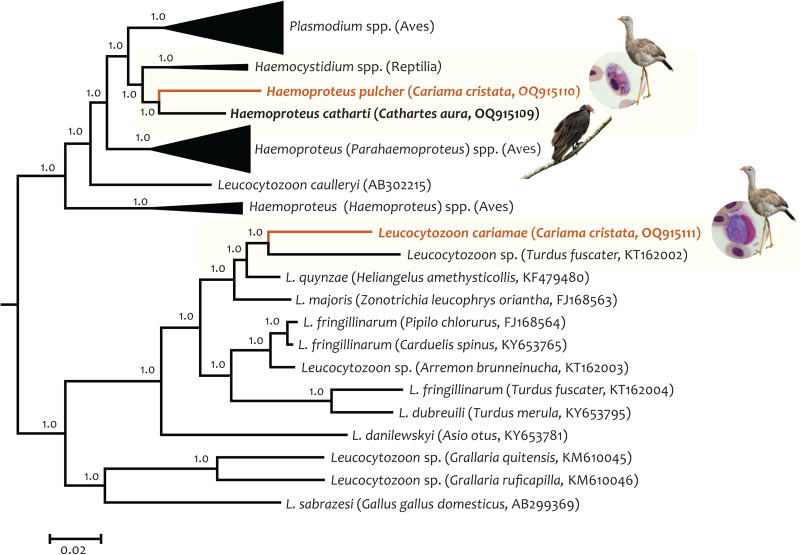
Bayesian phylogenetic hypothesis of haemosporidian parasites infecting red-legged seriema (*C. cristata*) sampled in Brazil. The phylogenetic tree was computed based on 74 partial parasites mtDNA genomes (5096 bp excluding gaps) belonging to 4 genera. The values above branches are posterior probabilities. Species found in this study are shown in orange, and the light-yellow boxes indicate their respective clade. GenBank accession numbers (as deposited in the MalAvi database) and their hosts are provided in parentheses for the sequences used in the analysis. More details about the species included in this analysis can be found in Supplementary Fig. S1.

Given that a comprehensive phylogenetic analysis using partial *cytb* gene sequences was already published in 2022 (see Fig. 1 in Carvalho *et al*., 2022), here we focus on the Bayesian phylogenetic hypothesis using the nearly complete mtDNA genomes (Fig. 3; Supplementary Fig. S1). The parasite with the closest relationship to *L. cariamae* n. sp. that has been sequenced at the mtDNA genome level is *Leucocytozoon* sp. TFUS14 (genetic distance = 0.101 ± 0.005), which was detected in a great thrush (*Turdus fuscater*, GenBank acc. no. KT162002) from Colombia. These 2 *Leucocytozoon* parasites, together with *Leucocytozoon quynzae* (from *Heliangelus amethysticollis* sampled in Colombia, KF479480) and *Leucocytozoon majoris* (from *Zonotrichia leucophrys oriantha* sampled in North America, FJ168563) are part of a monophyletic group that share a common ancestor with a clade of diverse *Leucocytozoon fringillinarum* lineages (from several hosts; Fig. 3) and with *L. dubreuili* (*Turdus merula*, KY653795). The mtDNA genome average genetic distances for these 2 clades are shown in Table 2.

**Table 2.** Estimates of pairwise genetic distance using nearly complete mtDNA genomes among haemosporidian parasites

		Genetic distance and standard error estimate(s)																	
	Parasite species	1	2	3	4	5	6	7	8	9	10	11	12	13	14	15	16	17	18
1	***L. cariamae* (OQ915111)**		**0**.**005**	0.004	0.004	0.005	0.005	0.005	0.006	0.006	0.006	0.008	0.008	0.007	0.007	0.008	0.008	0.008	0.008
2	*Leucocytozoon* sp. (KT162002)	**0**.**101**		0.004	0.004	0.005	0.005	0.005	0.006	0.006	0.006	0.007	0.008	0.007	0.008	0.007	0.007	0.007	0.007
3	*Leucocytozoon quynzae* (KF479480)	0.062	0.074		0.003	0.003	0.003	0.004	0.005	0.005	0.005	0.007	0.007	0.006	0.007	0.007	0.007	0.006	0.007
4	*Leucocytozoon majoris* (FJ168563)	0.069	0.085	0.039		0.004	0.004	0.004	0.005	0.005	0.006	0.007	0.007	0.007	0.007	0.007	0.007	0.007	0.007
5	*Leucocytozoon fringillinarum* (FJ168564)	0.097	0.100	0.058	0.063		0.001	0.002	0.004	0.004	0.005	0.007	0.007	0.007	0.007	0.007	0.007	0.007	0.007
6	*L. fringillinarum* (KY653765)	0.095	0.100	0.056	0.063	0.005		0.002	0.004	0.004	0.005	0.007	0.007	0.007	0.007	0.007	0.007	0.007	0.007
7	*Leucocytozoon* sp. (KT162003)	0.096	0.100	0.059	0.066	0.019	0.017		0.004	0.004	0.005	0.007	0.007	0.007	0.007	0.007	0.007	0.007	0.007
8	*L. fringillinarum* (KT162004)	0.135	0.127	0.104	0.108	0.089	0.089	0.090		0.003	0.007	0.008	0.007	0.007	0.008	0.007	0.007	0.007	0.007
9	*Leucocytozoon dubreuili* (KY653795)	0.130	0.124	0.098	0.104	0.089	0.088	0.086	0.053		0.006	0.008	0.008	0.007	0.008	0.007	0.007	0.007	0.008
10	*Leucocytozoon danilewskyi* (KY653781)	0.142	0.151	0.113	0.130	0.122	0.121	0.121	0.155	0.148		0.007	0.007	0.007	0.008	0.008	0.008	0.008	0.008
11	*Leucocytozoon* sp. (KM610045)	0.187	0.192	0.165	0.174	0.174	0.173	0.175	0.196	0.193	0.197		0.004	0.007	0.008	0.007	0.007	0.007	0.007
12	*Leucocytozoon* sp. (KM610046)	0.190	0.192	0.165	0.173	0.172	0.171	0.172	0.189	0.192	0.201	0.089		0.007	0.007	0.007	0.007	0.007	0.007
13	*Leucocytozoon sabrazesi* (AB299369)	0.176	0.179	0.150	0.159	0.162	0.162	0.163	0.175	0.169	0.192	0.162	0.164		0.007	0.007	0.007	0.007	0.007
14	***Haemoproteus pulcher* (OQ915110)**	0.174	0.183	0.157	0.164	0.170	0.170	0.170	0.187	0.185	0.196	0.173	0.175	0.178		**0**.**004**	0.004	0.004	0.004
15	*Haemoproteus catharti* (OQ915109)	0.169	0.175	0.156	0.161	0.162	0.163	0.166	0.178	0.177	0.191	0.167	0.165	0.169	**0**.**068**		0.004	0.003	0.004
16	*Haemocystidium* sp. (MT684458)	0.175	0.181	0.158	0.162	0.162	0.163	0.164	0.178	0.178	0.188	0.167	0.164	0.163	0.077	0.066		0.003	0.004
17	*Haemocystidium* sp. (MT684458)	0.170	0.181	0.150	0.157	0.158	0.158	0.162	0.177	0.175	0.190	0.169	0.164	0.160	0.077	0.063	0.055		0.003
18	*Haemocystidium* sp. (MT684460)	0.183	0.188	0.162	0.167	0.168	0.169	0.170	0.188	0.190	0.200	0.174	0.172	0.174	0.085	0.073	0.063	0.045	

The percentage of base substitutions per site between sequences and their standard error estimate(s) are shown below and above the diagonal, respectively. Analyses were conducted using the Tamura–Nei model. Estimates for those parasites found in *C. cristata* and its closely related parasite are shown in bold.

*Haemoproteus pulcher* from the seriema and the *H. catharti* from the turkey vulture, for which mtDNA sequence we provided here, are sister taxa (genetic distance = 0.068 ± 0.004) and shared a common ancestor with *Haemocystidium* parasites infecting Reptilia in South America, Africa and Australasia (Pacheco *et al*., 2020; Fig. 3, Supplementary Fig. S1). These 2 clades shared a common ancestor with parasites belonging to the genus *Plasmodium*. The mtDNA genome average genetic distances among *H. pulcher*, *H. catharti* and *Haemocystidium* spp. are shown in Table 2.

## Discussion

Our study is the first to provide morphological evidence of *Leucocytozoon* parasites infecting birds outside highland areas in the Neotropics. Because the host is a non-migratory species, it strongly supported that leucocytozoids are transmitted in the region. The first detection of a leucocytozoid infecting Cariamiformes birds enabled us to propose this newly discovered parasite as *L. cariamae* n. sp.

The presence of few forms of *L. cariamae* n. sp. on the slides we analysed (1 parasite detected during the examination of 200 microscopic fields at 1000× magnification) agrees with the general low parasitaemia observed in natural *Leucocytozoon* infections (Chagas *et al*., 2023; Valkiūnas and Iezhova, 2023). Indeed, microscopy alone underestimates the prevalence of haemosporidian parasites (Pacheco *et al*., 2022). However, this technique informs about host competence *via* visualization of gametocytes in blood smears (Valkiūnas and Iezhova, 2023), making it an important tool in avian Haemosporida research. Future studies evaluating seriemas with higher parasitaemia and displaying early stages of *L. cariamae* n. sp. are desirable to determine the type of blood cell utilized by the parasite described here. This parasitological trait may have phylogenetic value (Chagas *et al*., 2023) and should be further investigated.

We observed a coinfection between *Leucocytozoon* and *Haemoproteus* by using different diagnostic methods. *Haemoproteus pulcher* was not detected by the commonly used PCR protocol targeting the mtDNA *cytb* developed by Hellgren *et al*. (2004), as reported by Vanstreels *et al*. (2022), while *L. cariamae* n. sp. was not detected using the protocol described by Pacheco *et al*. (2018*a*, 2018*b*) to target the same gene. Although costly, the use of several PCR protocols combined with microscopy should be favoured in avian haemosporidian studies (Feldman *et al*., 1995; Jarvi *et al*., 2002; Richard *et al*., 2002; Valkiūnas *et al*., 2008; Braga *et al*., 2011; Clark *et al*., 2014).

The Bayesian phylogenetic analysis performed with nearly complete mitochondrial genomes (Fig. 3; Supplementary Fig. S1; Table 2) showed that *L. cariamae* does not share a recent common ancestor with the *fringillinarum* group. This supports our morphological findings and previous phylogenetic results obtained using the *cytb* gene fragment (Carvalho *et al*., 2022). The closest *Leucocytozoon cytb* lineage (GenBank acc. no. KX832566) has 96.45% similarity to *L. cariamae* n. sp. and was found in a host native to the western Palaearctic, from an avian order (Columbiformes) phylogenetically distant from the Cariamiformes (Jarvis *et al*., 2014; Prum *et al*., 2015; see Fig. 1 in Carvalho *et al*., 2022). Because *Leucocytozoon* diversity is relatively underreported compared to parasites belonging to the genera *Plasmodium* and *Haemoproteus* (Fecchio *et al*., 2021; Valkiūnas and Iezhova, 2023), further Haemosporida biodiversity research is needed to uncover species and/or genetic lineages closely related to *L. cariamae* n. sp. Specifically, it would be of notable interest to analyse the other member of the Cariamiformes, the black-legged seriema (*Chunga burmeisteri*), for the presence of haemosporidians.

Two *Leucocytozoon* morphospecies with the highest similarity to *L. cariamae* n. sp. parasitize birds from different orders and display morphological differences in relation to the new species described here. *Leucocytozoon podargii* infects Caprimulgiformes birds (tawny frogmouths) in the Australasian region (Adlard *et al*., 2002; Jiang *et al*., 2019). This parasite displays a granular cytoplasm, about half of the gametocytes were found in host cells without a remaining nucleus and microgametocytes are present in a very low proportion (Adlard *et al*., 2002). These characters are not observed in *L. cariamae* n. sp. Another closely related morphospecies, *L. californicus*, has been found in *F. sparverius* in California, USA (Walther *et al*., 2016). This bird species shares its habitat with seriemas in the Brazilian territory, and belongs to the Falconiformes order, a sister group of Cariamiformes (Jarvis *et al*., 2014; Prum *et al*., 2015). However, *L. californicus* exhibits characteristics not observed in *L. cariamae* n. sp.: the host cell nucleus is often positioned above the gametocyte and microgametocytes are strongly appressed to the host cell nuclei (Walther *et al*., 2016). None of these morphospecies have infected cells showing a small gap between the microgametocyte and the host nucleus (Fig. 1i–l), which seems to be a distinguishing feature of *L. cariamae* n. sp.

Our morphological evidence that *Leucocytozoon* parasites are transmitted in Brazil emphasizes that future studies should elucidate the diversity of competent vertebrate hosts in the Neotropics at low- and mid-elevation areas. Furthermore, evaluating Neotropical species of black flies for the presence of *Leucocytozoon* is another urgent task as this was explored only once (Lotta *et al*., 2016). According to Merino *et al*. (2008), Cuevas *et al*. (2019) and Fecchio *et al*. (2019), latitude was the most influential factor for the distribution of parasites of the genus *Leucocytozoon*, but altitude and local climate are also important variables to be considered (Greiner *et al*., 1975; Lotta *et al*., 2016; Cuevas *et al*., 2019). This would explain why the higher prevalence of leucocytozoids in the Neotropics would be more restricted to mountainous areas. Although *Leucocytozoon* development within black flies may be constrained by the year-round high temperature in some areas of the Neotropics (Fecchio *et al*., 2019), our results confirm a competent host in the Brazilian Cerrado, a requirement to sustain local transmission.

Considering that we initially screened 20 000 blood cells and found a single *L. cariamae* n. sp. gametocyte among high numbers of *H. pulcher* gametocytes, we recommend that future studies targeting *Leucocytozoon* in Neotropical areas conduct thorough blood smear analyses to avoid missing infections with such low parasitaemia. This has been reported in other haemosporidian parasites (Pacheco *et al*., 2022). This will also avoid underestimating possible coinfections, a common feature in *Leucocytozoon* infections (Chagas *et al*., 2023). Molecular approaches targeting *Leucocytozoon* parasites have been applied only recently in population- and community-wide studies in Brazil (Fecchio *et al*., 2018, 2019; Anjos *et al*., 2021; Carvalho *et al*., 2022); making evident the historical under-sampling of parasites of the genus in the Neotropics. Therefore, future haemosporidian studies in Neotropical areas should include parasites of the genus *Leucocytozoon* in their molecular (preferably using several primer sets that amplify not only the partial *cytb* gene but also the mtDNA genome) and microscopy analyses.

The *H. pulcher* detected here displays some morphological differences in relation to the original description, likely representing inter-population morphological variation within this parasite species. Additionally, differences such as the presence or absence of volutin granules, which can vary among samples collected from the same host species at the same location (Valkiūnas, 2005; Ferreira-Junior *et al*., 2018), may be due to variations in blood smear preparation (Valkiūnas, 2005). Differences in the mensuration of the morphological characters and/or microscope calibration should also be taken into account since most measurements (10 out of 12) were greater in the parasite described here, and because the length and width of uninfected erythrocyte nuclei also varied between our study and the original description (Vanstreels *et al*., 2022). However, this can also be explained by variations in erythrocyte morphology between birds from different populations (Haas and Janiga, 2020). Therefore, the morphological characters described here should be considered in the taxonomy of *H. pulcher*.

*Haemoproteus pulcher* was the first haemosporidian to be fully described in *C. cristata* (Vanstreels *et al*., 2022) and was detected in our study using the nested-PCR protocol by Pacheco *et al*. (2018*a*). Previous phylogenetic analysis using the *cytb* fragment placed this parasite and *H. catharti* in an unresolved position in a clade with a low posterior probability (pp = 0.52) between *Haemocystidium* spp. and *Plasmodium* spp. (see Fig. 2 in Vanstreels *et al*., 2022). However, the phylogenetic tree obtained with nearly complete mitochondrial genomes indicated, with high node supports (pp = 1.0), that *H. pulcher* and *H. catharti* form a monophyletic group that shares a common ancestor with the *Haemocystidium* clade found in reptiles globally (Pacheco *et al*., 2020).

*Haemocystidium* parasites produce microscopically visible haemozoin pigment and do not produce erythrocytic meronts in peripheral blood, traits that are shared with *Haemoproteus* parasites (González *et al*., 2019). This underscores molecular and morphological convergence among the *Haemocystidium* and *Haemoproteus* parasites. However, based on similar morphological aspects of tissue meronts between *Plasmodium* and *Haemocystidium*, this genus has been considered part of the family Plasmodiidae instead of Haemoproteidae (Telford, 1996). The fact that the clade containing *H. pulcher*, *H. catharti* and lizard *Haemocystidium* shares a common ancestor with the species belonging to the genus *Plasmodium* (Fig. 3; Supplementary Fig. S1) indicates this as a viable hypothesis. Our study shows that *H. pulcher* and *H. catharti* are not closely related to *H.* (*Parahaemoproteus*) spp. and *H.* (*Haemoproteus*) spp., corroborating findings by Galen *et al*. (2018) based on sequencing data from multiple nuclear loci showing that *H. catharti* is not closely related to Haemoproteidae parasites. *Haemoproteus* is a polyphyletic group (Pacheco and Escalante, 2023), and whereas solving this taxonomic issue will require additional work, Vanstreels *et al*. (2022) proposed that *H. pulcher* and *H. catharti* may constitute a new genus to be described. Based on our phylogenetic data at the mitochondrial genome level, placing these parasites in the genus *Haemocystidium* should be explored in future taxonomic revisions of Haemosporida when more mtDNA genomes become available for these groups of parasites.

## Conclusions

Despite the challenge of detecting the parasite due to the typically low intensity of parasitaemia in natural infections, we successfully identified and described a new species of *Leucocytozoon*. The description of *L. cariamae* n. sp. adds morphological evidence that leucocytozoids are transmitted outside highland areas in the Neotropics and characterizes red-legged seriemas as a competent host for this parasite group. The robust phylogenetic analysis presented here provides evidence at the mitochondrial genome level that *H. pulcher* and *H. catharti* are more closely associated with the reptile parasites *Haemocystidium* and with bird *Plasmodium* than with other bird-infecting *Haemoproteus*. This finding supports the polyphyly of the genus *Haemoproteus* or may indicate that *H. pulcher* and *H. catharti* might in fact compose the genus *Haemocystidium*. These hypotheses have broad implications for our understanding of the evolutionary relationships within Haemosporida.

## Supporting information

Vieira et al. supplementary material 1 Vieira et al. supplementary material

Vieira et al. supplementary material 2 Vieira et al. supplementary material

Vieira et al. supplementary material 3 Vieira et al. supplementary material

## Data Availability

The mtDNA genome sequences obtained in this study are deposited on GenBank under accession numbers OQ915109 (*Haemoproteus catharti*), OQ915110 (*Haemoproteus pulcher*) and OQ915111 (*Leucocytozoon cariamae* n. sp.).

## Supplementary material

The supplementary material for this article can be found at https://doi.org/10.1017/S0031182023000811
