# Supplementary material for: Leucocytozoon cariamae n. sp. and Haemoproteus pulcher coinfection in Cariama cristata (Aves: Cariamiformes): first mitochondrial genome analysis and morphological description of a leucocytozoid in Brazil
Source: Parasitology. 2023 Sep 1;150(14):1296–306. doi: 10.1017/S0031182023000811 (PMC10941214; doi:10.1017/S0031182023000811)
Supplement: Vieira et al. supplementary material 2 — Vieira et al. supplementary material [file S0031182023000811sup002.docx]

**Supplementary Fig. 1.** Expanded Bayesian phylogenetic hypothesis of haemosporidian parasites infecting Red-legged Seriema (*Cariama cristata*) sampled in Brazil. The phylogenetic tree was computed based on 74 partials parasites mtDNA genomes (5,096 bp excluding gaps) belonging to four genera. The values above branches are posterior probabilities. Species found in this study are shown in orange, and the light-yellow boxes indicate their respective clades. All Genbank accession numbers (as deposited in the MalAvi database) and their hosts are provided in parenthesis for the sequences used in the analysis.
