# Supplementary material for: Leucocytozoon cariamae n. sp. and Haemoproteus pulcher coinfection in Cariama cristata (Aves: Cariamiformes): first mitochondrial genome analysis and morphological description of a leucocytozoid in Brazil
Source: Parasitology. 2023 Sep 1;150(14):1296–306. doi: 10.1017/S0031182023000811 (PMC10941214; doi:10.1017/S0031182023000811)
Supplement: Vieira et al. supplementary material 3 — Vieira et al. supplementary material [file S0031182023000811sup003.docx]

**Supplementary Table 1.** Morphometric parameters of gametocytes of *Haemoproteus pulcher* and host cells of Red-legged Seriema (*Cariama cristata*). Sample size was 20 for all measurements.

| Feature Mean Range s.d. | | | |
| --- | --- | --- | --- |
| **Uninfected erythrocyte** |  |  |  |
| Length | 13 | 12.0–14.6 | 0.7 |
| Width | 7.6 | 6.7–8.9 | 0.6 |
| Length of nucleus^a^ | 6.2 | 4.9–7.4 | 0.6 |
| Width of nucleus^a^ | 3 | 2.7–3.6 | 0.2 |
| **Erythrocyte parasitized by macrogametocyte** |  |  |  |
| Length^a^ | 14 | 12.6–15.9 | 0.8 |
| Width^a^ | 8.4 | 7.5–9.3 | 0.5 |
| Length of nucleus^a^ | 5.7 | 4.8–6.8 | 0.7 |
| Width of nucleus | 2.8 | 2.4–3.3 | 0.2 |
| **Erythrocyte parasitized by microgametocyte** |  |  |  |
| Length | 13.6 | 11.9–15.3 | 1 |
| Width^a^ | 8.3 | 7.5–9.2 | 0.5 |
| Length of nucleus^a^ | 5.8 | 4.6–7.4 | 0.6 |
| Width of nucleus | 2.8 | 1.7–3.5 | 0.4 |
| **Macrogametocyte** |  |  |  |
| Length | 12.8 | 10.8–14.7 | 0.8 |
| Width^a^ | 3.7 | 3.0–4.4 | 0.4 |
| Length of nucleus^a^ | 3.1 | 2.3–4.0 | 0.5 |
| Width of nucleus | 3 | 1.5–3.8 | 0.5 |
| Nuclear displacement ratio | 0.6 | 0.3–0.8 | 0.1 |
| Number of pigment granules | 22 | 19-29 | 2.6 |
| **Microgametocyte** |  |  |  |
| Length | 11.5 | 9.9–12.7 | 0.7 |
| Width^a^ | 3.5 | 3.0–4.2 | 0.4 |
| Length of nucleus^a^ | 6.3 | 4.4–7.5 | 0.7 |
| Width of nucleus | 2.5 | 1.4–3.3 | 0.5 |
| Nuclear displacement ratio^a^ | 0.6 | 0.4–0.8 | 0.1 |
| Number of pigment granules^a^ | 15.9 | 12–21 | 2.0 |
|  |  |  |  |

**^a^** Statistically significant differences between measurements reported here and those for the original description of *H. pulcher* (Vanstreels *et al*., 2022).
